# poRe: an R package for the visualization and analysis of nanopore sequencing data

**DOI:** 10.1093/bioinformatics/btu590

**Published:** 2014-08-29

**Authors:** Mick Watson, Marian Thomson, Judith Risse, Richard Talbot, Javier Santoyo-Lopez, Karim Gharbi, Mark Blaxter

**Affiliations:** ^1^Edinburgh Genomics, The Roslin Institute and R(D)SVS, University of Edinburgh, Easter Bush, Edinburgh EH25 9RG and ^2^Edinburgh Genomics, Institute of Evolutionary Biology, Ashworth Laboratories, University of Edinburgh, Edinburgh EH9 3JT, UK

## Abstract

**Motivation:** The Oxford Nanopore MinION device represents a unique sequencing technology. As a mobile sequencing device powered by the USB port of a laptop, the MinION has huge potential applications. To enable these applications, the bioinformatics community will need to design and build a suite of tools specifically for MinION data.

**Results:** Here we present poRe, a package for R that enables users to manipulate, organize, summarize and visualize MinION nanopore sequencing data. As a package for R, poRe has been tested on Windows, Linux and MacOSX. Crucially, the Windows version allows users to analyse MinION data on the Windows laptop attached to the device.

**Availability and implementation:** poRe is released as a package for R at http://sourceforge.net/projects/rpore/. A tutorial and further information are available at https://sourceforge.net/p/rpore/wiki/Home/

**Contact:** mick.watson@roslin.ed.ac.uk

**Supplementary information**: Supplementary data are available at *Bioinformatics* online.

## 1 INTRODUCTION

Relative to first- and second-generation sequencing technologies, single-molecule sequencing is a new science, with only Helicos (Bowers *et al.*, 2009), Pacific Biosciences (Eid *et al.*, 2009) and Oxford Nanopore (ONT) being widely available. Even within the field of single-molecule sequencing, ONT’s nanopore sequencing technology represents a new paradigm; while both Helicos’ and Pacific Biosciences’ sequencing technologies measure incorporation events into a second strand, ONT’s MinION and GridION systems measure a single molecule of DNA as it passes through a protein nanopore. In addition, the MinION is the world’s first mobile DNA sequencer; it is powered by a laptop’s USB port and measures ∼4 inches in length. Recently, ONT opened up the MinION access programme, enabling researchers to use the device for the first time.

The ultra-low-cost and mobile nature of the MinION device opens up a huge number of applications. However, users of the device are faced with a number of informatics challenges. Users of the MinION must buy a high-specification Windows laptop, and thus there is a need for Windows-based software to handle the data. The MinION outputs binary files in the HDF5 format (http://www.hdfgroup.org/HDF5/). These contain raw data from the sequencer, which are then processed by a cloud-based base caller called ‘metrichor’. The subsequent called sequence files are also in HDF5 format (with the extension .FAST5). It is not uncommon for users to be presented with 30–50 000 HDF5 files (.fast5), with no software with which to access the data. Furthermore, data from all runs are stored in a single directory, with no subdirectories, and users find themselves needing to manipulate thousands of files manually, which takes time and is error prone.

We have developed poRe, a package for the statistical package R (http://www.R-project.org/; R Core Team, 2014), which enables users to manipulate MinION FAST5 files into run folders, extract FASTQ, gather statistics on each run and plot a number of key graphs, such as read-length histograms and yield-over-time. Crucially, as a package for R, poRe is cross-platform and has been tested on Windows, Linux and MacOSX. The Windows version enables users to run poRe on the MinION laptop itself, rather than copying the data to a Linux server to process with Perl or Python. This key feature brings users closer to true mobile DNA sequencing.

## 2 METHODS

### 2.1 Data format

The FAST5 HDF5 files contain a number of hierarchical groups, datasets and attributes, and these are described in more detail in the Supplementary Information.

### 2.2 Organization and run statistics

The first task users face is to organize a single MinION folder, which may contain reads from many different runs. We provide the function *copy.runs()* to help with this. The function reads all FAST5 files within a user-defined directory and extracts both the unique run identifier (‘run_id’) and the name and version of the base caller. Each read is then copied to a user-defined destination folder, under subfolders defined by the run_id and the name and version of the analysis. The latter is key, as each raw read may be base called many times by different versions of the metrichor base caller.

Embedded within each FAST5 file are a number of key statistics about the reads. These can be extracted for all reads in a run by the function *read.fast5.info()*. This returns a data frame with 24 columns of metadata for each read. The function *run.summary.stats()* can be used to extract key summary statistics, such as maximum, minimum and mean read lengths.

### 2.3 FASTQ and FASTA extraction

Once the data are organized, users may wish to extract FASTQ/A data. This can be done using the *extract.run.fastq()* and *extract.run.fasta()* functions. For each FAST5 read in a given directory, this function will extract the template, complement and 2D FASTQ/A data where they exist and write these to individual FASTQ/A files.

### 2.4 Data exploration

We provide a number of functions that allow users to explore the data visually. Histograms of read length can be created using the *plot.length.histogram()* function. This plots histograms for the template, complement and 2D read lengths (Supplementary Fig. S1).

The *plot.cumulative.yield()* function can be used to plot cumulative yield of the run over time, and sums up the template, complement and 2D read lengths over time in seconds since the analysis began (Supplementary Fig. S2).

Finally, the MinION device consists of a number of channels, each of which should contain a single nanopore. Users can count and plot the number of reads per channel for a run, using *plot.channel.reads()*, and sum and plot the yield per channel, using *plot.channel.yield().* Both of these can be potentially used to diagnose problems in particular areas of the flowcell.

### 2.5 Extracting and plotting events

**Fig. 1.**
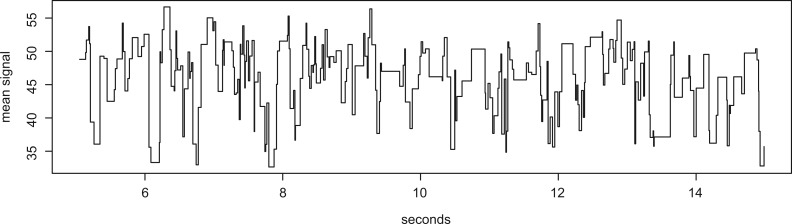
An example output from the *plot.squiggle* function. Plotted are the events data extracted from a single read. The *y*-axis is the mean electronic signal reported for the pore, and the *x*-axis is time in seconds

The raw data from the MinION are the information about the electronic signal measured as each single molecule of DNA passes through the protein nanopore. It is these data that are converted to sequence data by the metrichor agent. However, the raw events data are also available and can be extracted using the function *get.events()*. This will extract the thousands of events for both the template and complement for a particular read. The events data may then be visualized using the *plot.squiggle()* function (see Fig. 1).

## 3 DISCUSSION

We have written poRe, an R package that enables users to more easily manipulate, summarize and visualize MinION nanopore sequencing data. As a package for R, poRe is available for both Windows and Linux, and crucially the Windows version will allow data analysis on the mandatory Windows laptop on which the MinION depends. In addition, R is now a popular statistical package among biologists, who may feel comfortable using poRe through the R user interface.

poRe is one of the first bioinformatics packages to offer this necessary functionality. poretools (Loman and Quinlan, 2014), a toolkit written in Python, offers similar functionality, although each software has a different set of (overlapping) functions. A table comparing feature sets is available in the Supplementary Information. The cross-platform nature of poRe, its ease of installation and poRe’s ability to organize folders of FAST5 files make poRe an important tool for users of the MinION device.

## Supplementary Material

Supplementary Data
