# Supplementary material for: poRe: an R package for the visualization and analysis of nanopore sequencing data
Source: Bioinformatics. 2014 Aug 29;31(1):114–5. doi: 10.1093/bioinformatics/btu590 (PMC4271141; doi:10.1093/bioinformatics/btu590)
Supplement: Supplementary Data [file supp_31_1_114__index.html]

poRe: an R package for the visualization and analysis of nanopore sequencing data — poRe: an R package for the visualization and analysis of nanopore sequencing data — poRe: an R package for the visualization and analysis of nanopore sequencing data — Supplementary Data 

# poRe: an R package for the visualization and analysis of nanopore sequencing data

## Supplementary Data

files

**Files in this Data Supplement:**

- Supplementary Data - pdf file
