# Supplementary material for: poRe: an R package for the visualization and analysis of nanopore sequencing data
Source: Bioinformatics. 2014 Aug 29;31(1):114–5. doi: 10.1093/bioinformatics/btu590 (PMC4271141; doi:10.1093/bioinformatics/btu590)
Supplement: Supplementary Data [file supp_btu590_Supplementary_Data_V2.pdf]

Supplementary Data

Data format

The fast5 HDF5 files contain a number of hierarchical groups, datasets and attributes. An example structure is:

/Analyses
    /Basecall_2D_000
        /BaseCalled_2D
        /BaseCalled_template
        /BaseCalled_complement
        /Configuration
        /HairpinAlign
        /Summary
    /EventDetection_000
        /Reads
/Sequences
/UniqueGlobalKey

The raw events reported by each nanopore, as well as fastq data called by the metrichor base-caller, are embedded within the /Analyses/Basecall_2D_000 group, whereas meta-data about the run itself is embedded within the /UniqueGlobalKey group.

Additional Figures

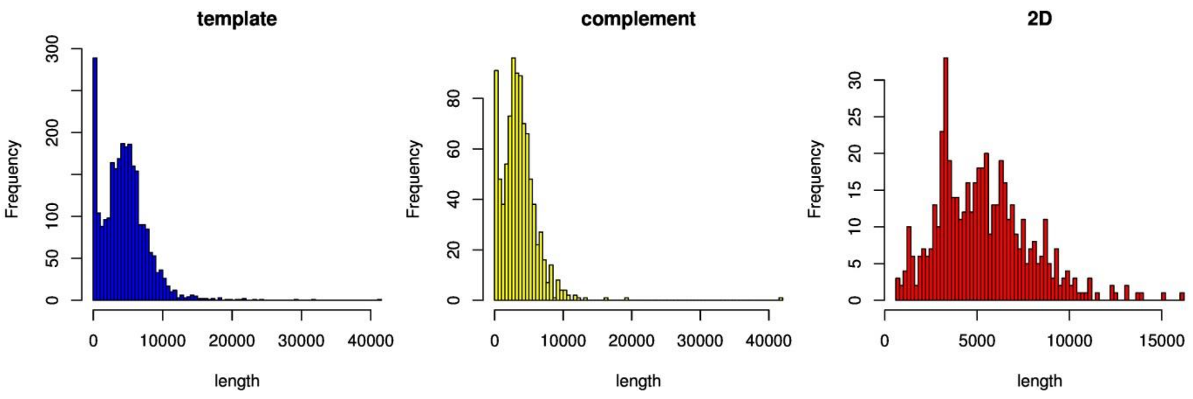

Figure S1. Read length histograms from the *plot.length.histogram* function, showing the distribution of read lengths from the template, complement and 2D reads

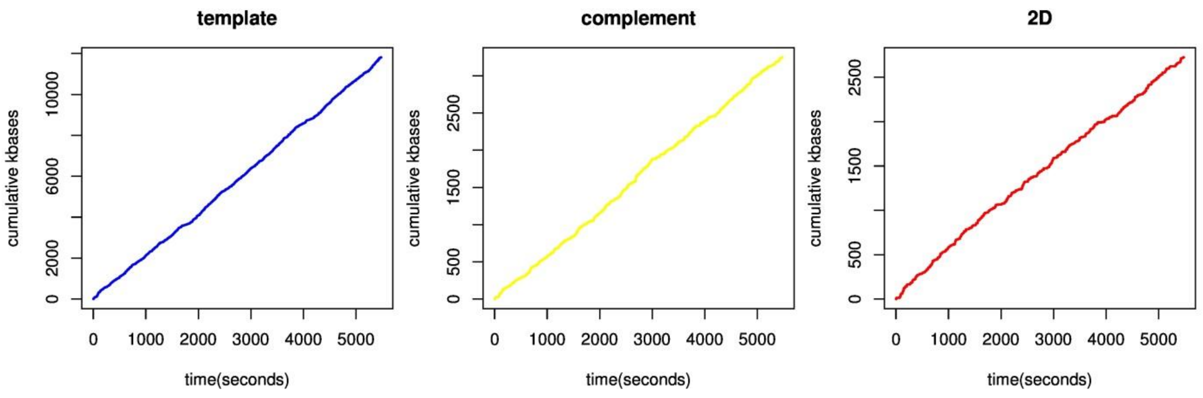

Figure S2. Cumulative yield plot from the *plot.cumulative.yield* function. The y-axis is time, and the x-axis is cumulative yield in kilobases (kb) for the template, complement and 2D reads

Feature comparison with poretools

Feature	poRe	poretools
Extract fastq	Y	Y
Extract fasta	Y	Y
Organise fast5 into run folders	Y	
Create tar files of runs		Y
Plot yield	Y	Y
Plot squiggle	Y	Y
Extract run stats	Y	Y
Find longest read		Y
Read length histogram	Y	Y
Nucleotide distribution		Y
Quality score distribution		Y
Extract events data	Y	Y
Extract channel statistics	Y	
Plot channel statistics	Y	
